# Supplementary material for: A high-resolution mRNA expression time course of embryonic development in zebrafish
Source: eLife. 2017 Nov 16;6:e30860. doi: 10.7554/eLife.30860 (PMC5690287; doi:10.7554/eLife.30860)
Supplement: Supplementary file 6. [file elife-30860-supp6.zip › biolayout-clusters-files/Cluster238-genes.html]

Cluster238


# Cluster238: Genes

| Ensembl ID | Gene Name | Chr | Start | End | Biotype |
| --- | --- | --- | --- | --- | --- |
| ENSDARG00000079305 | hbae3 | 3 | 54891985 | 54893256 | protein\_coding |
| ENSDARG00000089963 | hbbe1.1 | 3 | 54859451 | 54877454 | protein\_coding |
| ENSDARG00000055514 | icn2 | 16 | 23572019 | 23576061 | protein\_coding |
| ENSDARG00000052917 | si:ch211-202f3.3 | 17 | 45594074 | 45615938 | protein\_coding |
| ENSDARG00000100431 | spaca4l | 19 | 17451427 | 17459867 | protein\_coding |
| ENSDARG00000101200 | zgc:112964 | 12 | 43922560 | 43939050 | protein\_coding |
